# Nanoparticle formation utilizing simple polyacrylic acid-cation coacervates as template

**DOI:** 10.1039/d6ra00330c

**Published:** 2026-02-12

**Authors:** Bastian Rödig, Patrick Denk, Ulrich Schürmann, Matthias Kellermeier, Werner Kunz

**Affiliations:** a Institute of Physical and Theoretical Chemistry, University of Regensburg, Universitätsstr. 31 D-93053 Regensburg Germany; b Faculty of Engineering, Kiel University Kaiserstr. 2 D-24143 Kiel Germany; c Material Science, BASF SE, RGA/BM-B007 Carl-Bosch-Str. 38 D-67056 Ludwigshafen Germany

## Abstract

The liquid–liquid phase separation (coacervation) of simple solutions containing only polyacrylic acid (PAA) and multivalent transition metal (TM) cations can be used as a template for nanoparticle formation. It is shown that transition metal carbonate/sulfide/oxide nanoparticles can be prepared by a simple coacervate-mediated process using only PAA, the transition metal chloride, and either sodium carbonate or sulfide. The rather simple approach first demonstrated for calcium carbonate could be extended to the chosen transition metals Co, Mn, Ni, and Cu. Using DLS and UV/vis, the formation and properties of PAA/TM coacervates were studied showing that in a broad pH range, coacervation is possible when a critical cation concentration is reached. Using these findings, mineralization of the coacervates results in defined small nanoparticles that can be easily separated from other bigger residues. Calcination of carbonate particles results in their respective oxide counterparts. The rather small sizes (5 nm) of the primary particles and their amorphous crystal structure suggest further investigation into their use as catalysts.

## Abstract 2

Investigation on the formation of simple transition-metal polyacrylic acid coacervates and their use as a template for the synthesis of sulfide/carbonate/oxide nanoparticles.

## Graphical abstract

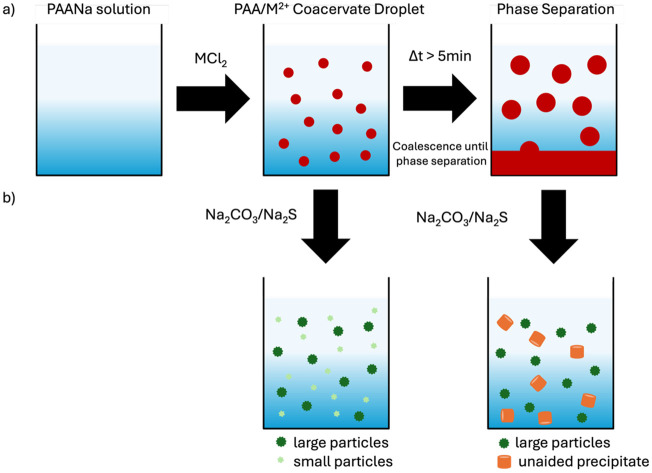

## Introduction

Research on nanoparticles is at an all-time high due to their role in numerous applications, ranging from catalysis^1^ and electronics^2^ to energy storage,^3^ sensors,^4^ and functional coatings.^5,6^ Several techniques have been established for the synthesis of nanoparticles with precise characteristics—such as sol–gel methods,^7^ hydrothermal^8^ and solvothermal techniques,^9^ and mechanochemical strategies^10^—achieving control over their size, composition, structural form (whether crystalline or amorphous), and surface properties. Despite all these achievements, the control over these parameters remains a challenge and the process is often elaborate. In contrast, a rather simple approach for the synthesis of amorphous calcium carbonate (ACC) using coacervates as a template is shown by Kaempfe *et al.*^11^

Coacervation is a special form of phase separation in colloidal systems, especially known for aqueous polyelectrolyte solutions. Coacervation was first mentioned by the Dutch chemists Hendrik Bungenberg de Jong and Hugo Kruyt, who investigated gelatin solutions.^12,13^ It is a form of liquid–liquid phase separation, in which a colloidal solution separates into a colloid-rich (coacervate) and colloid-poor (equilibrium) phase. These coacervates form spontaneously throughout the solution and, depending on the density and surface properties, the coacervate phase can remain dispersed in the equilibrium phase or coalesce to form a top or bottom phase.^12,14^ Coacervate formation depends on primary properties, such as solubility, molecular weight, charge density, and hydrophobicity of the sol as well as secondary properties primarily affecting the solution such as temperature, pH, salt concentration, and solvent.^14^ Changes in these parameters can lead to reduced solubility of the colloid and induce coacervation.^12,13^ Coacervation is a complex balance of electrostatics, hydrophobicity, excluded volume, van der Waals, and other contributions to the system's overall stability.^15^ Coacervates are typically divided into two classes. While simple coacervates only consist of one colloidal or colloid-forming species at certain environmental conditions, complex coacervates consist of at least two colloidal or colloid-forming, usually polymeric, surface-active compounds.^14^ Simple coacervate formation of polyelectrolytes depends on salt or alcohol concentration, or a change in pH or temperature.^16^ Generally speaking, a dehydrating agent (solvent, salt, or both) can cause simple coacervation by reducing the interaction between colloid and solvent, resulting in favorable inter-colloid interactions.

Scattering experiments and cryo-TEM measurements have shown the hollow, spongelike structure inside the coacervate droplets for various simple and complex coacervates,^17^ which makes them predestined as templates for nanoparticle synthesis. In previous works, polyacrylic acid (PAA)/Ca^2+^ coacervates were used to produce defined CaCO_3_ microstructures and nanoparticles,^11,18^ which is in this work extended to PAA/transition metal cation coacervates and respectively particles of transition metal sulfide/carbonate/oxide. It will be shown that the mineralization of these coacervates results in small particles of around 5 nm, which can be isolated from bigger structures forming in the supernatant.

## Experimental

### Materials

Poly(acrylic acid, sodium salt) (PAANa) solution (*M*_w_ ≈ 8000, 0,45 wt% in H_2_O, *M*_w_ ≈ 2000, 0,45 wt% in H_2_O), poly(acrylic acid) (PAA) solution (*M*_w_ ≈ 100 000, 0,35 wt% in H_2_O), cobalt(ii) chloride hexahydrate (purity ≥98%), manganese(ii) chloride tetrahydrate (BioReagent grade), nickel(ii) chloride (purity ≥98%), sodium carbonate (purity ≥99.5%), 1M HCl solution (for titrations) were purchased from Sigma Aldrich (St. Louis, USA). Copper(ii) chloride dihydrate (purity ≥99.0%) and sodium hydrosulfide monohydrate (purity ≥90%) were obtained from Honeywell (Charlotte, North Carolina, USA). All chemicals were used without further purification. Ultrapure water from a Millipore purification system (resistivity > 18 M Ω cm) was used for all systems containing water.

### Formation of coacervates and synthesis of particles

Aqueous solutions of PAANa were prepared from 1 to 10 wt% at different pH values ranging from 4 to 8.6 (native pH). The pH was adjusted using 1 M HCl solution. Aqueous solutions of 0.25 M transition metal chloride were prepared and added dropwise to the PAA solutions under continuous stirring with a magnetic stirrer until the onset of turbidity indicates coacervate formation. Mineralization was achieved by adding the stochiometric ([M^2+^] = [CO_3_^2−^/S^2−^]) amount of either sodium carbonate or sodium sulfide to the solution immediately after coacervate formation. By centrifugation, the formed particles were separated from the supernatant. After resuspension in water, they were lyophilized to obtain a dry powder. Oxides were formed by calcination of the respective carbonate powder at 600 °C for at least 6 h.

### Characterization

X-ray diffraction analysis was carried out using a STADI-P device from STOE & Cie GmbH (Darmstadt, Germany), with CuK_α1_ radiation (*λ* = 1.540598 Å). The dry samples were ground to form a homogeneous powder and scanned from 2° to 92° in 0.015° steps.

For dynamic light scattering (DLS) measurements, a CGS-3 Compact Goniometer System from ALV (Langen, Germany), combined with an ALV/LSE-5004 Multiple Tau Digital Correlator and a vertically polarized 22 mW HeNe laser (*λ* = 632.9 nm), was used. Measurements were performed at a scattering angle of 90° with a constant temperature of 25 °C for 300 s. Using the 2nd order cumulant analysis^19,20^ of the ALV-7004 Correlator software, the hydrodynamic radius and size distributions can be calculated from the autocorrelation function. Also, the CONTIN algorithm^21,22^ was used to gather information from more noisy measurements. Coacervates were measured immediately after formation (*cf.* Fig. S3). Particles were resuspended in water and briefly sonicated with an ultrasonic probe from Hielscher (Germany) before the measurement.

The structure and size of the particles were evaluated using transmission electron microscopy (TEM) using a CM-12 transmission electron microscope from FEI/Phillips (Dreieich, Germany) with a tungsten cathode and 120 kV acceleration voltage. Pictures were taken with the digital camera 1kx1k from TVIPS (Gauting, Germany). The images were processed and evaluated with the software EMMenu4. Samples were resuspended in EtOH and prepared on 200 mesh copper grids with a carbon film from PLANO GmbH (Wetzlar, Germany).

UV/vis measurements were carried out at the infinite m nano^+^ UV/vis photo spectrometer from TECAN (Männedorf, Switzerland) using a 96-well plate to determine cation concentration. Therefore, the supernatants of the coacervate samples were measured after a settling time of 24 h after full phase separation.

## Results and discussion

**Fig. 1:**
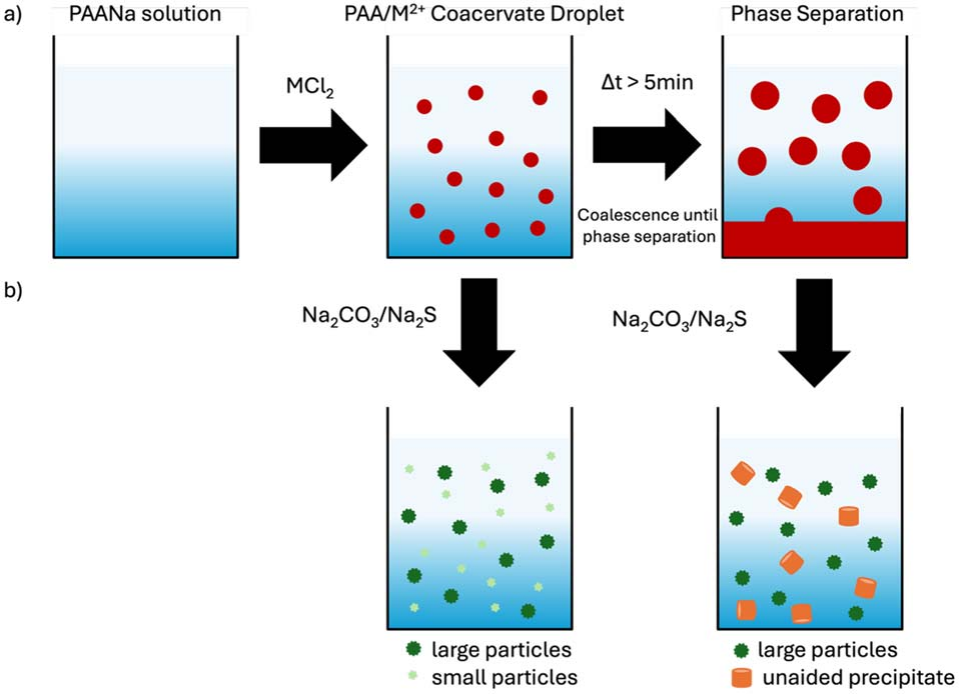
Scheme of the simple coacervating system: (a) PAA^−^/Co^2+^ coacervate droplets form at concentrations greater than a certain threshold ratio and coalesce over time. (b) Mineralization with Na_2_CO_3_/Na_2_S results in different structures before and after phase separation. (inspired by^11^).

Fig. 1a schematically shows the formation of coacervates by the addition of metal chloride solution (MCl_2_) to the PAANa solution. The formed coacervate droplets coalesce over time and ultimately, they fuse to form a separate phase. The supernatant is the polymer-depleted phase, whereas the lower phase contains a high amount of PAA^−^/M^2+^ coacervates and is therefore highly viscous.

Once formed, the separated phase cannot be resolubilized again, which also hinders the mineralization in the second step. Shown in Fig. 1b is the mineralization process at different times after the coacervate droplet formation. The simple coacervation formation of divalent cations and PAA is fundamental to the investigation of the mineralization process to form nanoparticles. Therefore, the association of divalent cation and PAA to form coacervate droplets and their phase behavior is studied first and the mineralization of the coacervates is shown afterward. While mostly results with Co^2+^ are shown, additionally Ca^2+^, Cu^2+^, Mn^2+^, and Ni^2+^ were tested. If not stated otherwise, *M*_W_ ≈ 8000 g mol^−1^ PAANa was used and the concentration in the final mixtures is 1 wt%.

### Coacervate formation

**Fig. 2:**
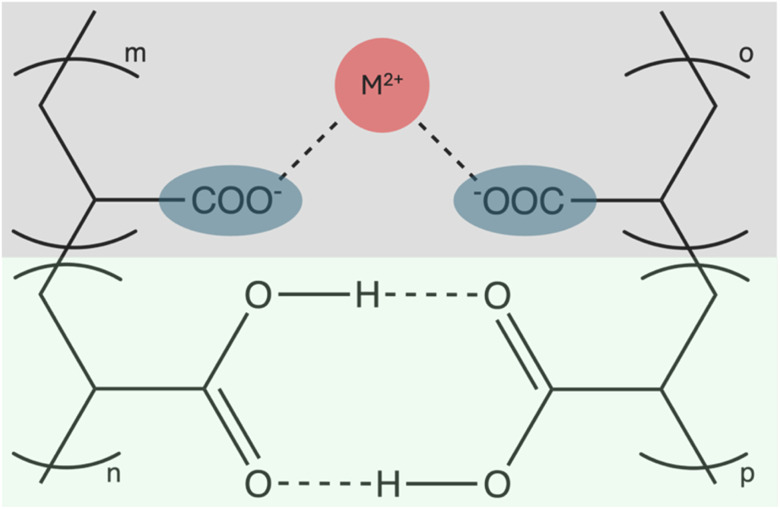
The two main attractive interactions to form simple PAA/M^2+^ coacervates. Divalent cation (M^2+^) is complexed by deprotonated carboxylic groups and can bridge between two PAA chains (highlighted gray). Protonated carboxylic groups can form hydrogen bonds between different PAA chains (highlighted green).

To form the coacervates, the two attractive interactions shown in Fig. 2 are essential. At elevated pH values the carboxylic groups on the PAA chain are deprotonated. The divalent cation can be complexed by the negatively charged carboxylic group and can, this way, bridge two different PAA chains (highlighted in grey). At lower pH values, the carboxylic groups are more protonated. In this state, the formation of hydrogen bonds between different PAA chains is possible (highlighted in green). The pH dependence of the fractional charge *f* of PAA is given by:^23^1
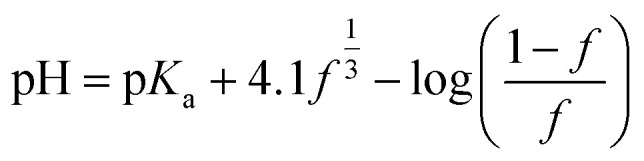


With p*K*_a_ as the acid dissociation constant. This interplay of different interactions results in a wide range of pH values, where the simple coacervation of PAA/M^2+^ is possible. The range indicates that a significant portion, around 3–4 of the carboxylic groups have to be protonated for sufficient attraction to form coacervates. pH titrations for various divalent cations (*cf.* Fig. S1) have shown that complexed cations do not precipitate as hydroxide. Instead, the coacervates are resolubilized again at a sufficiently high pH, resulting in the formation of a clear solution beyond the pH at which the hydroxide formation of the respective cation begins. This happens not only for cations like Cu^2+^, which forms soluble hydroxides at elevated pH values, but for all cations tested. So, the upper limit for coacervate formation is not the precipitation of metal hydroxide (M(OH)_2_), but the dissolution of the coacervates due to inadequate interactions, especially to few formed hydrogen bonds. Nevertheless, the competition of hydroxide formation and PAA/M^2+^ complexation occurs in certain pH ranges depending on the cation, increasingly important for hydroxides with smaller solubility products like Cu^2+^ (*cf.* Fig. S2).

**Fig. 3:**
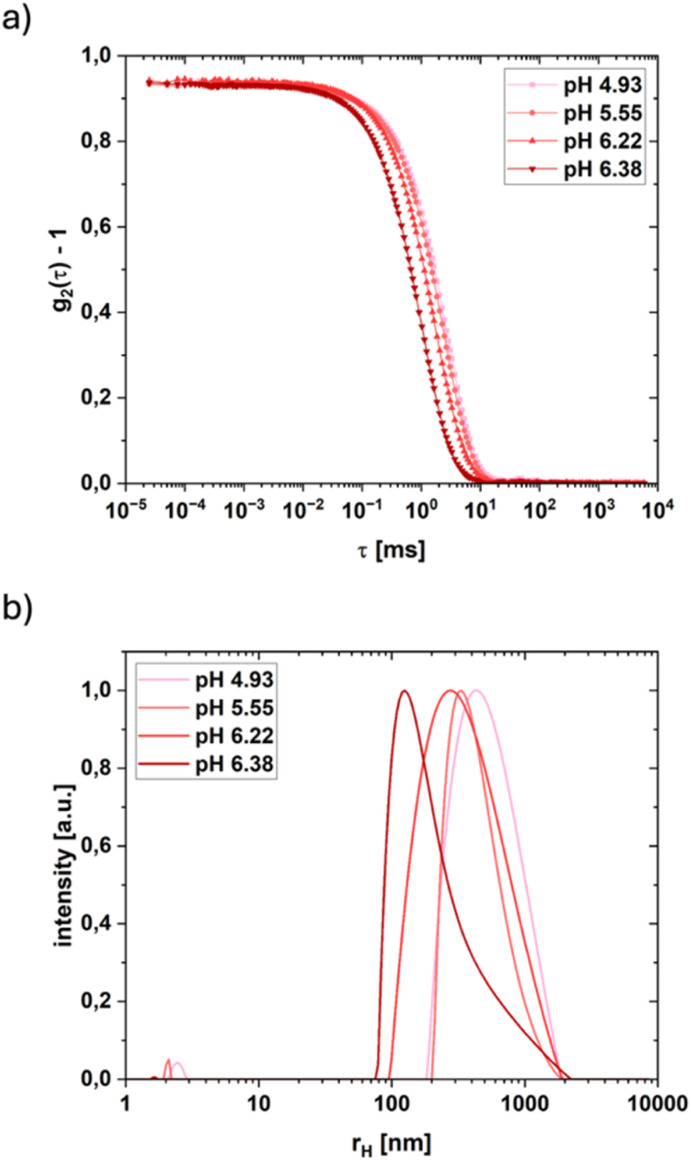
(a) DLS correlation function of PAA^−^/Co^2+^ coacervates formed at different pH values measured at 25 °C. Debye length with decreasing pH: 0.488 nm, 0.522 nm, 0.664 nm, 0.739 nm. (b) Intensity-weighted size distribution of PAA^−^/Co^2+^ coacervates at different pH values, derived from DLS using the cumulants analysis. Note that the samples are already subject to coalescence during measurement and should not be regarded as the absolute size of the coacervate droplets, but rather as an indication for different coalescence rates. Further information on coalescence rates can be found in Fig. S3.

The addition of divalent cation to a solution containing PAA in the right pH regime can result in coacervate formation, whereas simple pH titration or the addition of monovalent cations like Na^+^ does not (*cf.* Fig. S1). At the elevated end of the pH range, DLS measurements of the coacervate droplets, immediately after formation (*cf.* Fig. S3), indicate in smaller radii than for lower pH values, as visible in Fig. 3. The measurable size of the droplets depends on a balance of different forces. First, due to the higher charges of the PAA chains at higher pH, more cations can be complexed, resulting in more electrostatic attraction inside the coacervate phase and therefore the formed coacervate droplets are more compact. Secondly, at lower pH values, the coalescence is faster, due to fewer surface charges on the coacervate droplets and therefore less electrostatic repulsion between the droplets. Even though the Debye length λ_D_ (Fig. 3a) remains smaller than 1 nm for the investigated pH values, the small decrease with increased pH shows less screening at lower pH values which results, combined with fewer charges on the coacervate droplets, in faster coalescence. This is hardly observable for Ni^2+^ and Cu^2+^, because both induce the formation of PAA/cation flakes at higher pH values indicating a competition of complexation by the carboxylic groups and hydroxide precipitation. This results in a narrower pH range, in which coacervation is possible, but the droplets are already subject to fairly rapid coalescence. The solubility product of the respective hydroxides^25^ is lower compared to the other cations tested. Corresponding to the onset of hydroxide formation at this pH value, the required minimal copper concentration of around 24 mM is almost half of the concentration necessary for other cations (*cf.* Fig. S2). Especially for copper, coacervation is only possible at pH values lower than 4.8 whereas for nickel the respective pH value is around 7, resulting in coacervate formation at pH values below with increasing flocculation with increasing pH. At lower pH values, coacervation is possible until too less carboxylic groups are deprotonated to interact sufficiently with the cations. No coacervation was observed at pH values ≤ 4 or ≥ 7 for all tested cations.

**Fig. 4:**
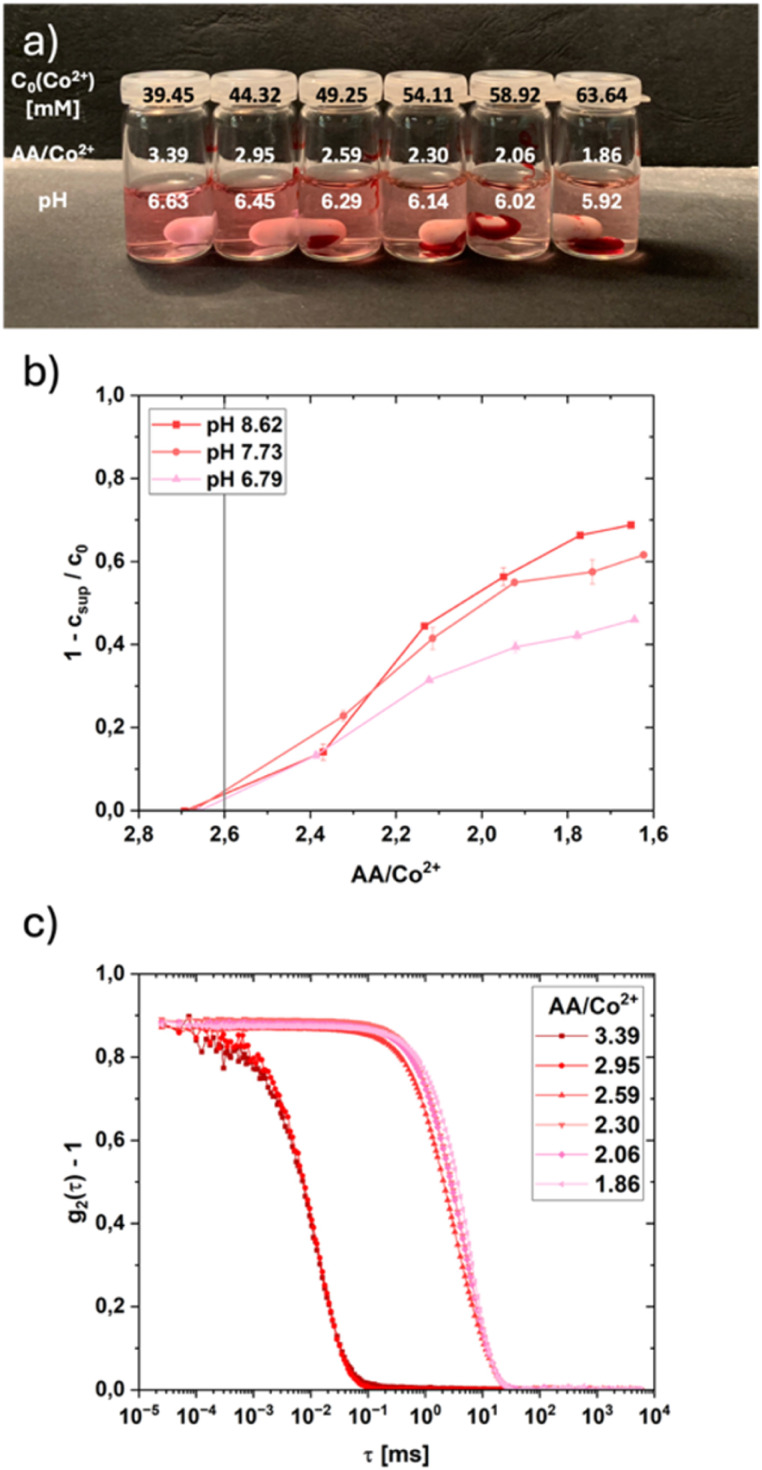
(a) Picture of PAA/Co^2+^ solutions with different AA/Co^2+^ ratios after an equilibration time of 24 h containing PAA/Co^2+^ coacervates after coalescence. (b) Share of the Co^2+^ concentration in the coacervates of the total concentration at the different AA/Co^2+^ ratios derived from UV/vis measurements of the supernatants after coalescence (equilibration time 24 h). (c) DLS correlation functions of PAA/Co^2+^ solutions at the different AA/Co^2+^ ratios (3.4–1.9), measured immediately after coacervate formation, when the coacervates were still finely dispersed as droplets (*cf.* Fig. S3).

To form coacervates a certain threshold concentration of divalent cation^14,26^ must be reached. This threshold depends on the PAA concentration, the pH of the solution,^24^ the chain length of the PAA,^24,27,28^ and to some degree also on temperature.^26^ The optimal chain length for the experiments was to be found at 8000 g mol^−1^. Longer chains needed a longer time to reach the equilibrium and often formed flakes during the addition of the cation, whereas the shorter chain coacervates coalesce faster and also showed a higher threshold concentration (1 wt% of 2000 g mol^−1^ PAANa, c(M^2+^) ≈ 80 mM, AA/M^2+^ ≈ 0.8) whereas the cation species showed minimal influence on the threshold concentration of around 40 mM (AA/M^2+^ ≈ 2.6) for most of the cations tested, Cu^2+^ behaves differently with a threshold concentration of around 24 mM (AA/Cu^2+^ ≈ 6.6). Below this concentration, association of divalent cation and PAA occurs, but the total interactions are not yet enough to result in a phase separation. If the concentration is raised beyond this threshold, coacervates form (*cf.* Fig. S1). The higher the concentration, the faster the coalescence to a unified phase, depleting the supernatant of the polymer as well as the cation. Visible in Fig. 4a is the increased volume of the separated coacervate phase and the fading color of the supernatant for higher concentrated samples. Visible in Fig. 4b are the Co^2+^ concentrations in the supernatants, as measured by UV/vis spectroscopy, revealing that the fraction of cations actively participating in coacervate formation increases with decreasing acrylic acid (AA)/M^2+^ ratio. From the onset of coacervation around an AA/M^2+^ ratio of 2.6, where only a small fraction of the cations are bound in the coacervate, the fraction of the cations bound in the coacervate increases to around 70% at the highest pH shown. Whereas the onset concentration remains the same for different pH values, the amount of bound cation decreases with decreasing pH. This is a direct result of less deprotonated carboxylic groups on the PAA chains resulting in fewer complexation sites for the cations. A similar trend is visible in Fig. S4a, where the total organic carbon (TOC) and therefore the PAA remaining in the supernatant solution decreases with increasing pH and increasing cobalt concentration. Overall, the decrease in the PAA content with increasing pH values is only strictly visible after the AA/M^2+^ ratio of 2.6 is undercut. Above, no pH dependence can be seen, but even in the samples where no coacervation is recognizable, only about 90% of the PAA can be detected *via* TOC measurements. At the highest cation concentration (65 mM) and the highest pH value (5.9) approximately 75% of the total PAA in solution is bound in the coacervate phase, which correlates with the amount of removed cation in the supernatant.

The calculation of the actual amount of interacting deprotonated carboxylates, determined using eqn (1), and the amount of cation in the coacervates, results in a ratio of 0.45 AA^−^/M2+ at the onset of coacervation at the highest pH value tested. At even lower ratios (AA/M^2+^ < 1.6), the concentration in the supernatant seems to slightly increase again hinting at an almost complete association of available PAA molecules and precipitation/phase separation as coacervates. Or in other words, the remaining PAA in the supernatant is not sufficient to form further coacervates despite sufficient cobalt concentration.

The onset of coacervation is also visible in Fig. 4c. For cation concentrations below the threshold concentration (≈40 mM, AA/Co^2+^ ≈ 2.6), the correlation function only represents dissolved PAA molecules at a size of around 2.2 nm. Above the threshold concentration, the correlation function represents the formed coacervate droplets and is shifted to slightly higher lag times, indicating bigger droplets, for higher cation concentrations. This is a result of faster coalescence and slightly smaller pH values. Whereas parts of the coacervate droplets near the threshold concentration remain dispersed for almost 24 h, the further away from the threshold concentration, the faster the coalescence. The additional amount of cation introduces more screening resulting in greater Debye lengths over the concentration series. The critical cation concentration for the onset of coacervation is independent of the pH value (adjusted with HCl) resulting in a constant ratio of AA/M^2+^. Therefore, the calculated AA^−^/M^2+^ decreases with a decrease in pH, indicating that less transition metal cations are complexed at lower pH values, which is directly visible in Fig. 4b.

### Mineralization of the coacervates

Depending on the time Na_2_CO_3_/Na_2_S is added, different structures will be created, as implied in Fig. 1b. Although previous results by Kaempfe *et al.*^11^ showed an increase in particle size with larger coacervate droplets, no direct correlation between particle size and coacervate droplet size could be established due to the rather fast coalescence of the droplets over time. Controlling particle size *via* coacervate size was therefore not investigated further here.

**Fig. 5:**
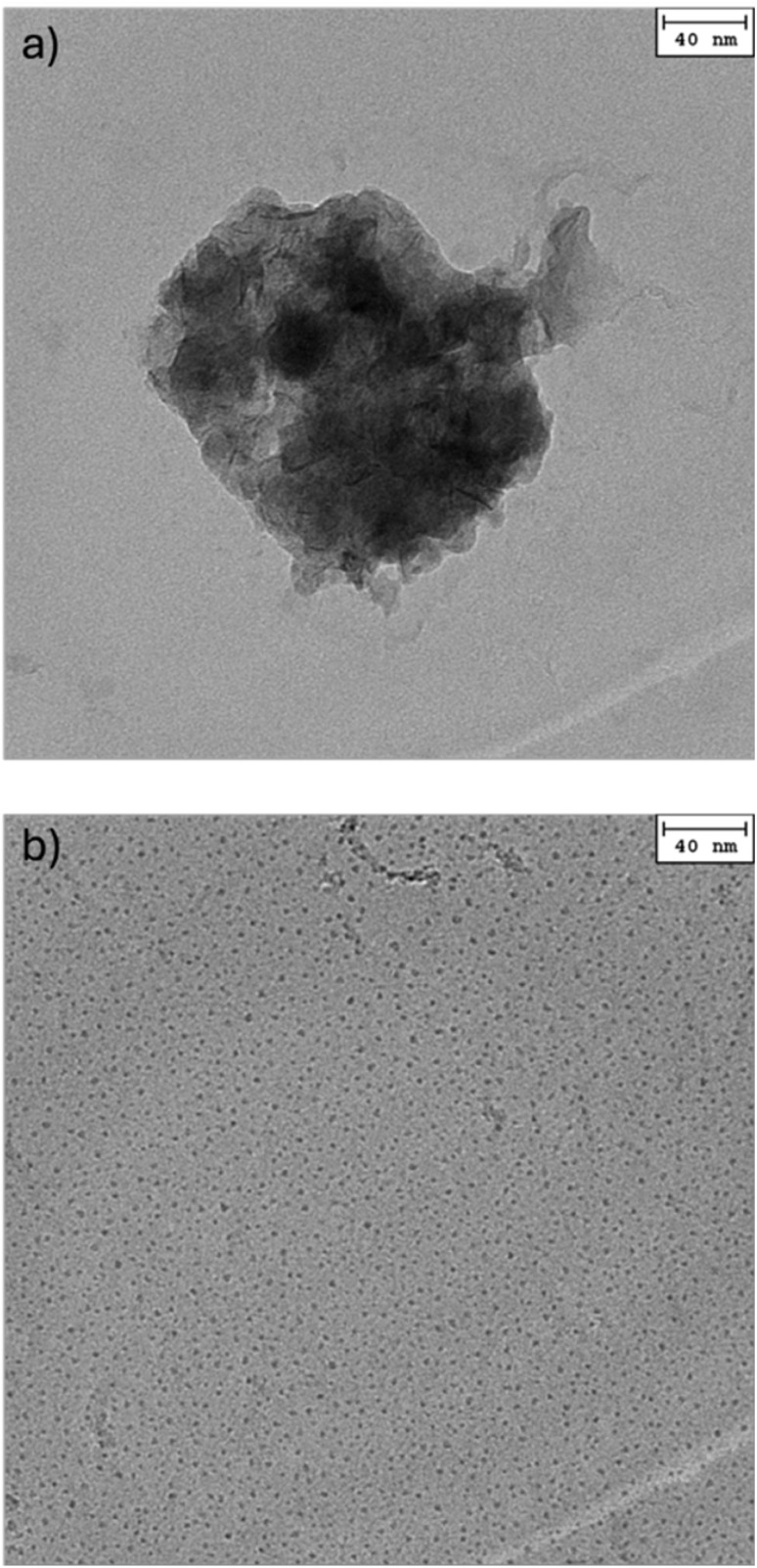
TEM micrograph of CoS particles derived from PAA^−^/Co^2+^ mixtures at different ratios. (a) AA/Co^2+^ ratio of 2.65, just below the threshold concentration. (b) AA/Co^2+^ ratio of 1.33, above the threshold concentration.

Instead, the mineralization was done immediately after the formation of the coacervates or, as different approach, only in the separated coacervate phase. Due to the insolubility and the highly viscous state of the separated coacervate phase, it is assumed that structures only formed by leaking cations into the supernatant and were not mediated by the coacervate phase itself. Some formed structures are shown in SI compared to structures derived from uncontrolled precipitation (*cf.* Fig. S5). The similarity of the found structures validates the unmediated growth of the particles in the supernatant. Synthesis of particles below (AA/M^2+^ >2.6) and beyond (AA/M^2+^ <2.6) the threshold concentration results in different structures, as visible in Fig. 5 for CoS and in Fig. S6 for CoCO_3_.

For concentrations below the threshold, big clusters of smaller particles that have grown together form (Fig. 5a), whereas at higher concentrations small particles, of a few nanometers in size surround aggregates of bigger particles (Fig. 5b). Even those aggregated particles are smaller than particles seen for particles derived from below the threshold concentration and still are distinguishable (Fig. 5b and S7–S9). This immense size reduction of particles comes from the reduced chemical potential of the bound Co^2+^ cations and therefore the greatly reduced reactivity. Even though the formation of the smallest particles is enhanced in the synthesis farther away from the threshold concentration, due to the higher amount of bound cations, synthesis above but still near the threshold results in an easier process and more reproducible particle sizes due to the slower coalescence of the coacervate droplets and therefore more homogeneous distribution of cation prior to synthesis. Also, higher pH values were preferred for the synthesis, first, to prevent excessive gas development from the added carbonate/sulfide, and second, to use the smallest possible coacervate droplets and reduce coalescence as much as possible. The amount of bound cations in the coacervates also is higher at higher pH values due to more ion bridging, as shown in Fig. 4b. The mineralization immediately after the addition of the MCl_2_ solution then results in a mixture of two or sometimes three distinguishable particle distributions (*cf.* Table S1). The different structures formed are shown in Fig. S7–S9 for the different cations, Co^2+^, Mn^2+^, and Ni^2+^. Visible in all samples, regardless of the cation or the anion, relatively big almost circular/spherical structures occur. These are direct remains of the synthesis and just represent the coacervate droplets (*cf.* Fig. S10). The measurable size correlates to the hydrodynamic radii derived from DLS measurements evaluated using the Cumulants method (*c.f.* Table S1). These particles also seem to be substructured and their poor contrast on the carbon film indicates that they consist mainly of similar electron-dense material as the background and be therefore just coacervate droplets. There are also two other structures visible. First small particles, only a few nanometers in size (5–15 nm), and second bigger particles in the range of 20–60 nm. The bigger particles occur also in other samples such as the particles derived from unaided precipitation (*cf.* Fig. S5c and d) and in the synthesis below the threshold concentration (*cf.*Fig. 5a) and reduce in number the further above the threshold concentration. Nevertheless, the most interesting particles are the smallest ones about 5 nm in size. These are visible in almost all samples but in different quantities (*cf.* Fig. S11). Generally, they appear more often the lower the AA/M^2+^ ratio is, until the saturation of cations in the coacervates, then the amount of big particles increases again. The evaluation of the DLS data with the CONTIN algorithm fits the measured sizes very well (*cf.* Table S1). Filtration using a 200 nm syringe filter or centrifugation (<700 g) can further decrease the polydispersity leading only to two distinct structures, small particles of a few nanometers and their aggregates (*c.f.* Table S1).^11^

**Fig. 6:**
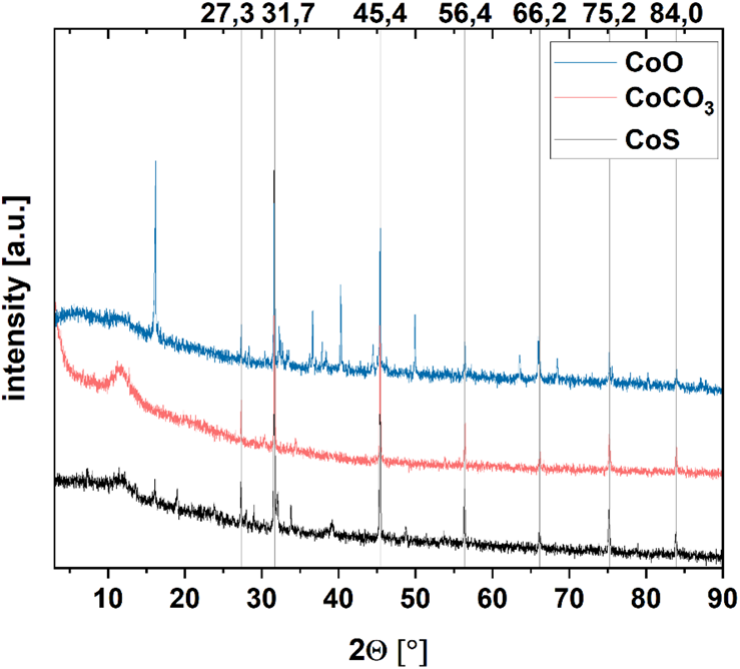
XRD measurement of Co-particles derived from PAA/Co^2+^ coacervates at pH 5.45 with different anions, CoS (black), CoCO_3_ (red), and CoO (blue). The reflexes visible and the reference lines correspond to the NaCl lattice derived from NaCl residues.

XRD measurements, shown in Fig. 6 indicate crystalline NaCl residues from the synthesis. Even though the concentration of the respective cobalt species is higher than for the residues, almost no clear reflexes can be detected except for CoO. Instead, a broad reflex at small angle is visible, hinting at amorphous crystal structures. Amorphous crystal structures were expected, as this was shown already before for ACC particles.^11^ In the high-resolution TEM images, the particles, which are only a few nanometers in size, appear to have a crystal structure that cannot be resolved due to their small size (*cf.* Fig. S11). The calcination of the carbonates to the oxides does not show the amorphous reflex anymore, but instead shows, depending on the calcination temperature, the respective crystal structure of the formed oxide. This indicates the rearranging of the atoms in the lattice resulting in a defined crystal structure, while maintaining the small size. This can also be seen for the other cations, Mn^2+^ and Ni^2+^(*cf.* Fig. S12). The amount of formed particles for Cu^2+^ is lower, compared to the other cations due to the necessity of lower pH values for the coacervate formation and therefore less bound cations in the coacervates. This is also visible in the XRD spectra of the Cu-containing particles (Fig. S11), as the amorphous structures are less expressed, and the evaluation of the DLS data results in only a small percentage of the smallest particles if even possible (*cf.* Table S1).

## Conclusions

Synthesis of small transition metal sulfide/carbonate/oxide nanoparticles in the range of a few nanometers (<10 nm) using coacervates as template is possible. Even though the formed suspension contains multiple structures the desired small particles can be isolated quite easily, either by filtration or centrifugation. Although the coacervation process differs slightly for various cations, especially pronounced for Cu^2+^, there are also some similarities. Increased cation concentrations beyond the threshold concentration result in accelerated coalescence.^29,30^ At too high pH or too high ionic strength, the coacervates are not able to form due to lack of attractive interactions, such as screened electrostatics or lack of hydrogen bonds.^29,31^ There are hints that an increased amount of cation decreases the amount of water in the formed coacervate and therefore increases the viscosity.^12,13^ Even though the influence of temperature was not tested, lower temperatures should lead to coacervates with a higher volume fraction and a higher polymer concentration.^32^ Whereas elevated temperatures increase the coalescence rate.^26^ Also, the chain length affects the onset of the coacervate formation. Shorter chains can form coacervates at lower ion concentrations.^29^ Even though the coacervate droplet size has only a minor influence on the produced particle size, the synthesis at elevated pH values seemed to be the most promising. The coacervates form smaller droplets and higher surface charges resulting in slower coalescence and sedimentation. The higher pH enables also most of the added carbonate/sulfide to precipitate with the cation instead of escaping as gas. A more deprotonated PAA can also complex more cation resulting in a higher yield of synthesized nanoparticles mediated by the coacervates instead of unsupported precipitation in the bulk media. The small size, resulting in a high surface area and the amorphous crystal structure, may enable applications as catalysts.

## Conflicts of interest

There are no conflicts to declare.

## Supplementary Material

RA-016-D6RA00330C-s001

## Data Availability

Data for this article are available at Publikationsserver der Uni Regensburg at https://epub.uni-regensburg.de/78141/1/Data.zip. Supplementary information (SI) is available. See DOI: https://doi.org/10.1039/d6ra00330c.
