# Supplementary material for: Nanoparticle formation utilizing simple polyacrylic acid-cation coacervates as template
Source: RSC Adv. 2026 Feb 12;16(10):8633–40. doi: 10.1039/d6ra00330c (PMC12896128; doi:10.1039/d6ra00330c)
Supplement: RA-016-D6RA00330C-s001 [file RA-016-D6RA00330C-s001.pdf]

Supplementary Information (SI) for RSC Advances.
This journal is © The Royal Society of Chemistry 2026

**Nanoparticle formation utilizing simple polyacrylic acid-cation coacervates as template**

Bastian Rödig[1], Patrick Denk[2], Ulrich Schürmann[4], Matthias Kellermeier[5] and Werner Kunz[3]

[1-3] University of Regensburg, Institute of Physical and Theoretical Chemistry, Regensburg, Germany

[4] Faculty of Engineering, Kiel University, Kaiserstr. 2, D-24143 Kiel, Germany

[5] BASF SE, Material Physics, Ludwigshafen, Germany

# Supplementary Information

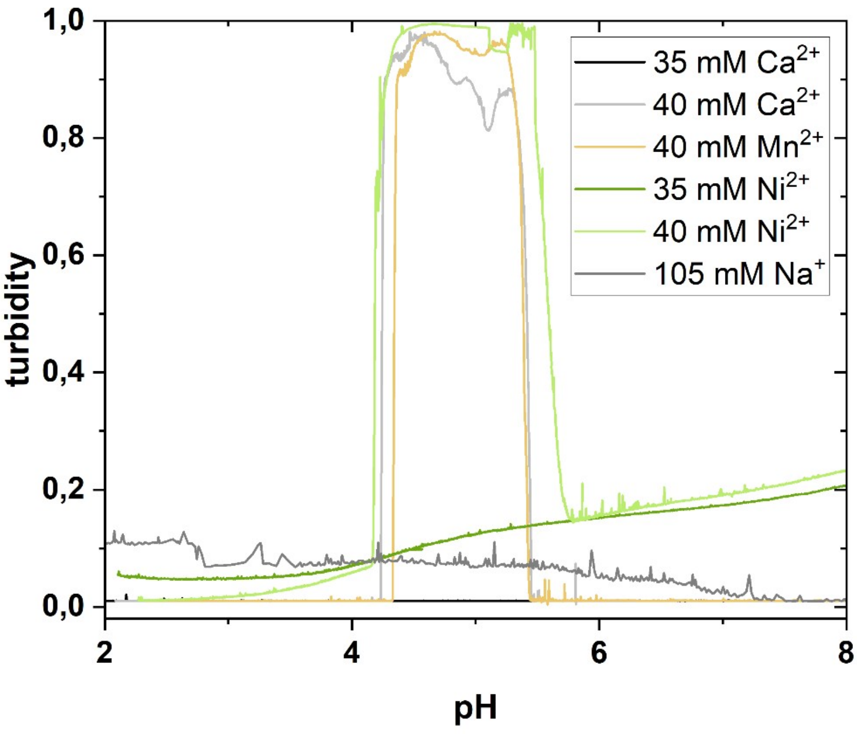


*Figure S1. Titration of a solution of 1w% 100k PAA / 40mM $MCl_2$ with 1M NaOH with continuous measurements of pH and turbidity at $\lambda$ = 660 nm. The concentration of PAA and $MCl_2$ was held constant by titrating a solution of 2w% 100k PAA /*

*80mM $MCl_2$ in the same volumetric amounts as the NaOH. Note that the increase in "turbidity" for $Ni^{2+}$ comes due to absorption of the AA/$Ni^{2+}$ complex at $\lambda$ = 660 nm.*

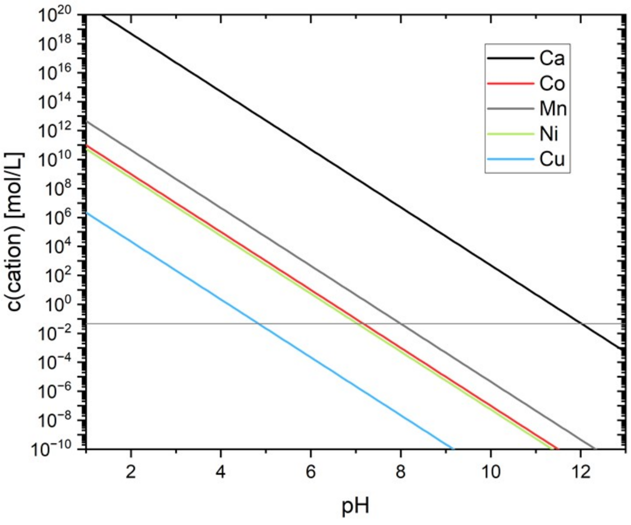


*Figure S2. Solubility products of the tested cation ($Ca^{2+}$, $Co^{2+}$, $Mn^{2+}$, $Ni^{2+}$, $Cu^{2+}$) hydroxides against the pH. The reference line indicates the threshold concentration of the cation for coacervate formation. Note that the threshold concentration for PAA/$Cu^{2+}$ coacervates is lower than for the others, and the reference line represents only the other cations. Extracted pH values are: pH($Ca^{2+}$) = 12, pH($Co^{2+}$) = 7.2, pH($Mn^{2+}$) = 8, pH($Ni^{2+}$) = 7, pH($Cu^{2+}$) = 4.8.*

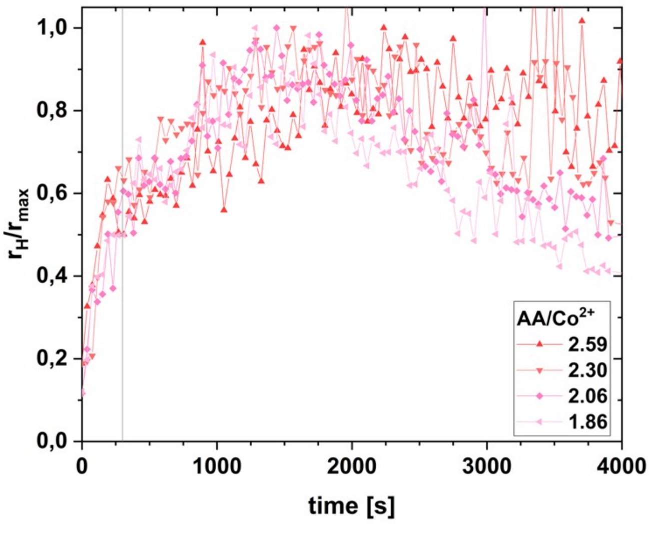


*Figure S3. Aggregation and coalescence/precipitation kinetic of freshly prepared Co-coacervates as shown in Figure 4c. Typical DLS-measurements were measured in the first 300s. Note that no coacervate sizes are given in this figure but a $r_H/r_{max}$ ratio to show the coalescence rates independend of actual size. Hydrodynamic radii ($r_H$) were determined using Cumulants analysis on DLS measurements of coacervate droplet suspensions at different AA/$Co^{2+}$ ratios every 30s for at least 1h. $r_{max}$ represents the maximum value for hydrodynamic radius observed in this timeframe. The increase in droplet size does not stagnate after 1000s but the sedimentation of the coalesced droplets removes the largest droplets from measurable solution, seemingly decreasing the droplet size again.*

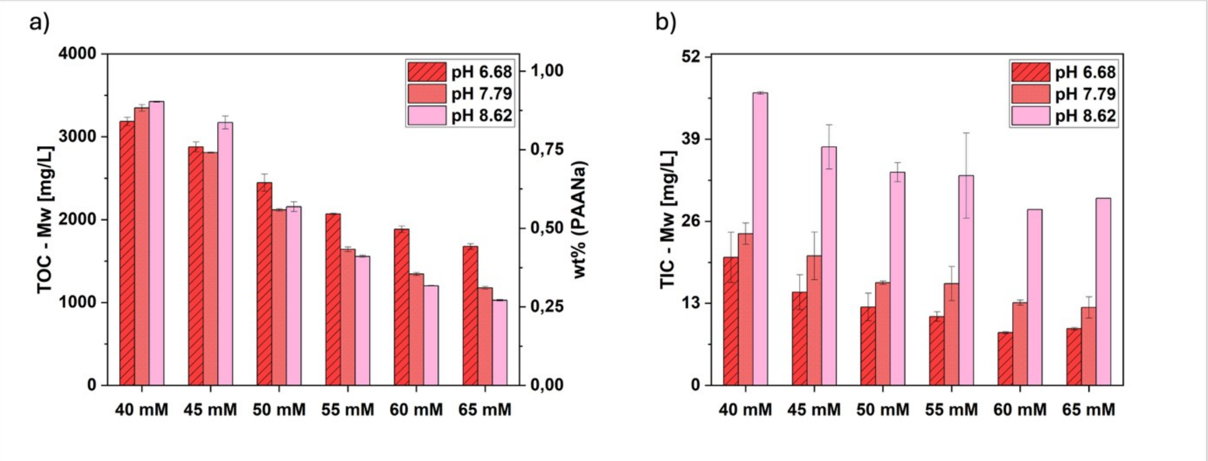


*Figure S4. Total organic carbon (a) and total inorganic carbon (b) of the supernatants shown in Figure 4a. Also visible in a is the calculated wt% of remaining PAA in the supernatant.*

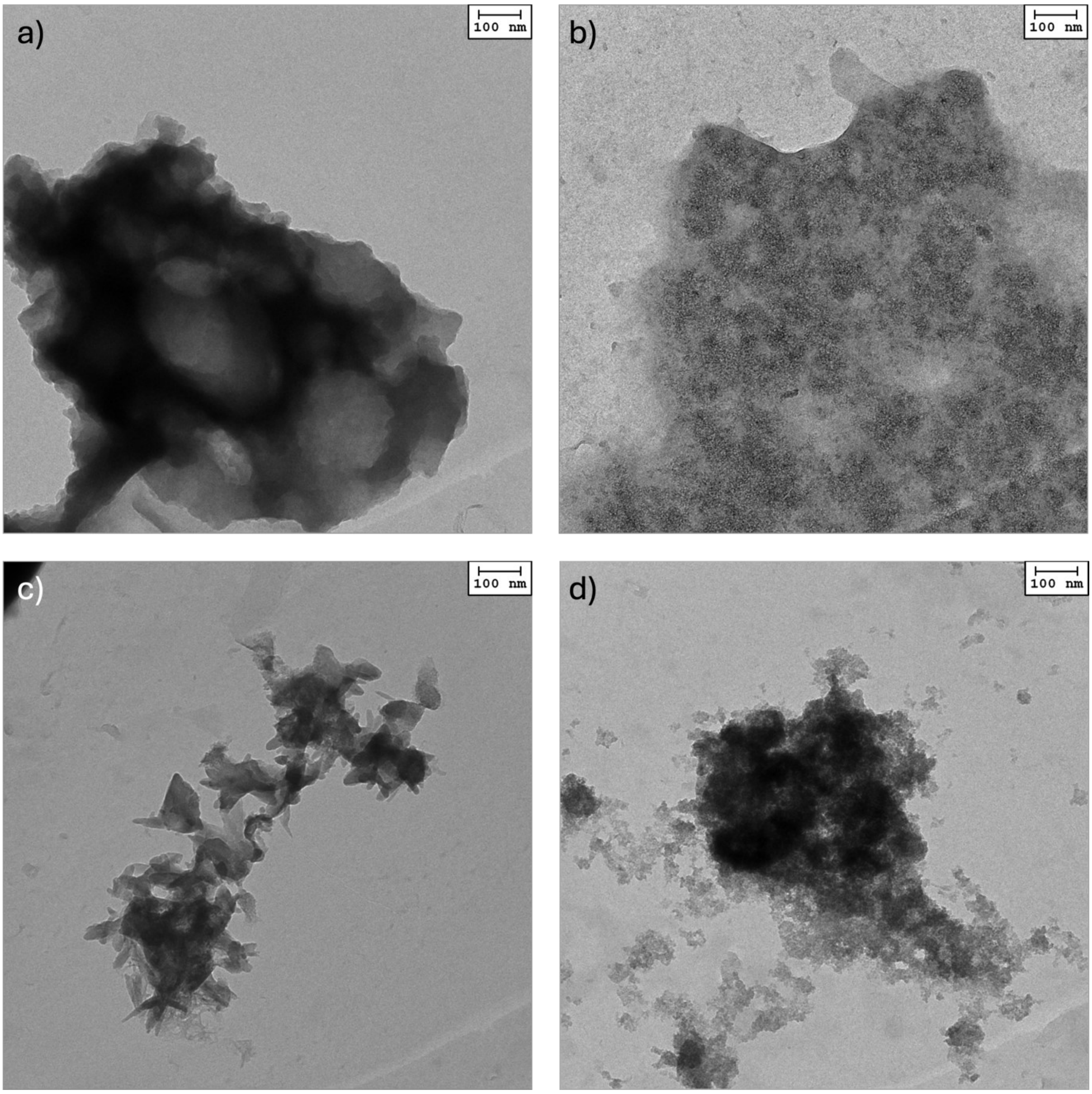


*Figure S5. TEM micrographs, at a magnification of 1:13000, of particles derived from $PAA^-/Co^{2+}$ coacervates at pH 5.45 (a) $MnCO_3$. b) CoS) and TEM micrographs of particles derived from unsupported precipitation of c) $CoCO_3$ and d) CoS.*

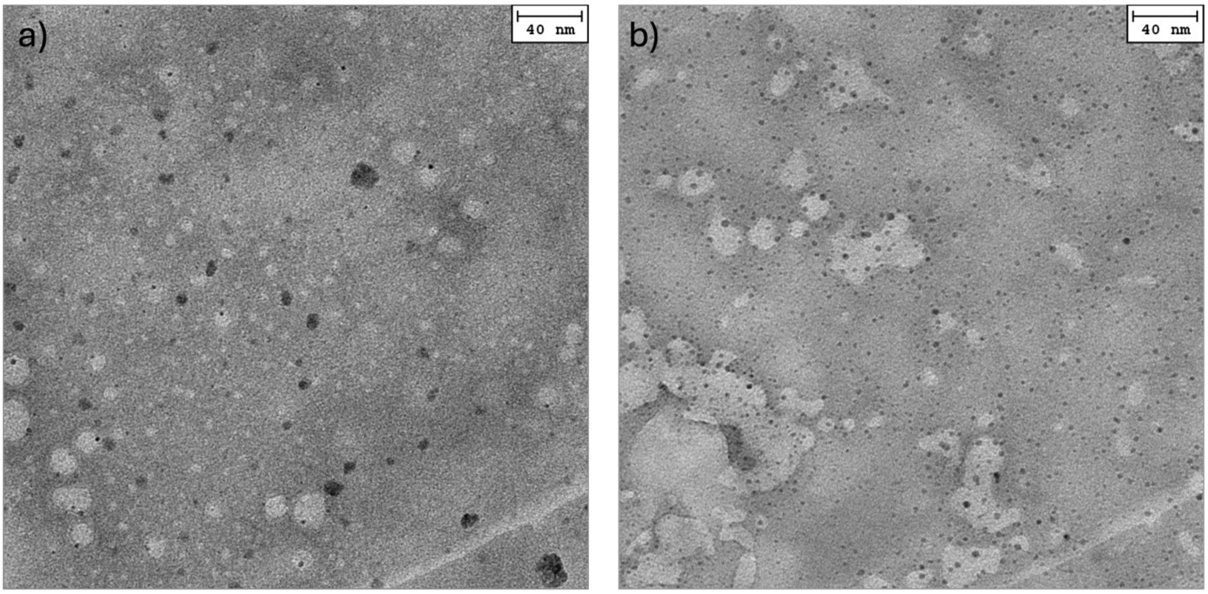


*Figure S6. TEM micrograph of $CoCO_3$ particles derived from $PAA^-/Co^{2+}$ mixtures at different ratios. a) $AA/Co^{2+}$ ratio of 2.65, just below the treshold concentration. B) $AA/Co^{2+}$ ratio of 1.33, way above the treshold concentration.*

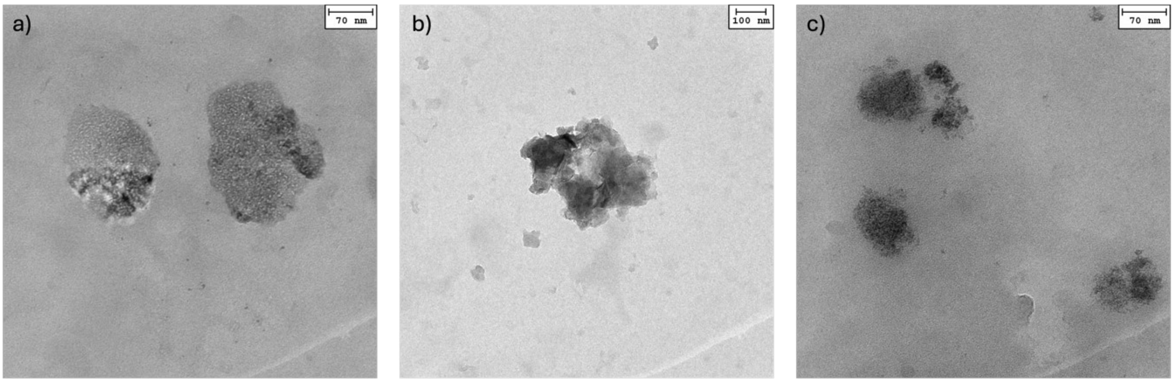


*Figure S7. TEM micrograph of particles derived from $PAA^-/Co^{2+}$ coacervates at pH 5.45. a) $CoCO_3$, 1:28000. b) CoO derived from $CoCO_3$ after calcination at 650°C, 1:13000. c) CoS, 1:28000.*

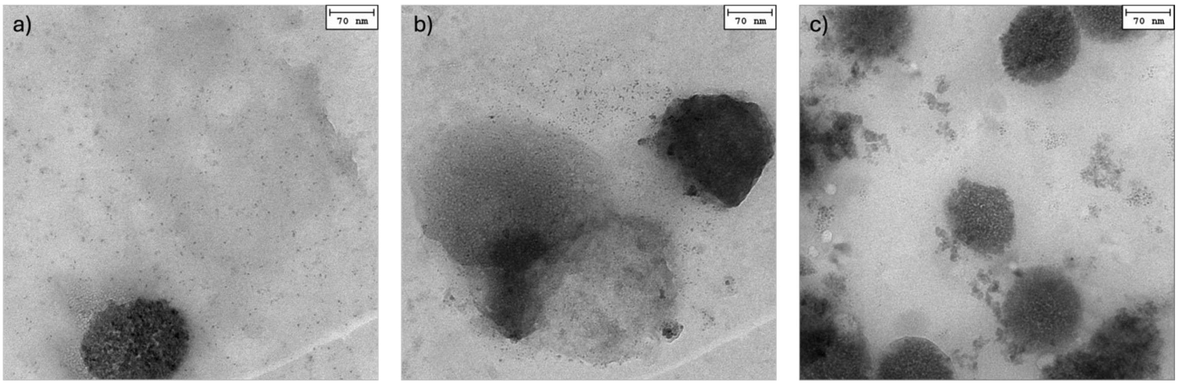


*Figure S8. TEM micrograph of particles derived from $PAA^-/Mn^{2+}$ coacervates at pH 5.45. a) $MnCO_3$. b) MnO derived from $MnCO_3$ after calcination at 650°C. c) MnS. Magnification of 1:28000.*

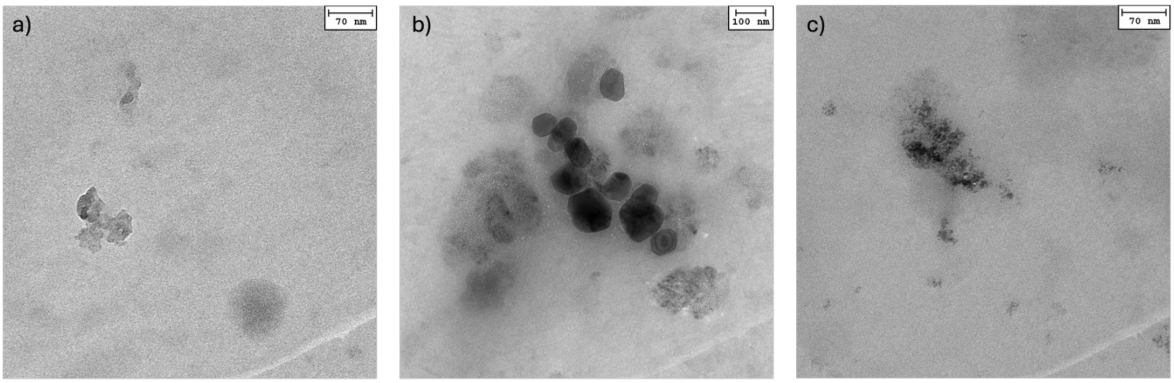


*Figure S9. TEM micrograph of particles derived from $PAA^-/Ni^{2+}$ coacervates at pH 5.45. a) $NiCO_3$, 1:28000. b) NiO derived from $NiCO_3$ after calcination at 650°C, 1:13000. c) NiS, 1:28000.*

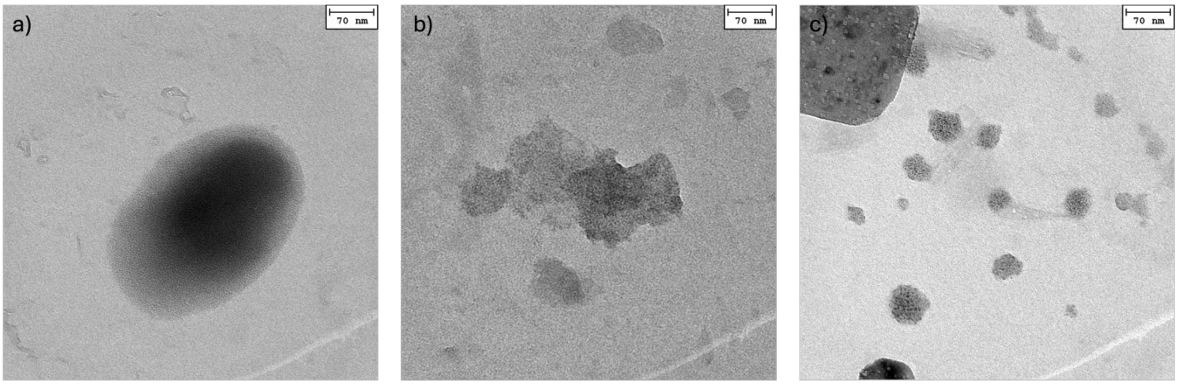


*Figure S10. TEM micrograph of different $PAA^-/M^{2+}$ coacervates at a magnification of 1:28000. a) M = Ca. b) M = Co. c) M = Cu, note that the cubic structure in the top left corner is NaCl residue left from synthesis.*

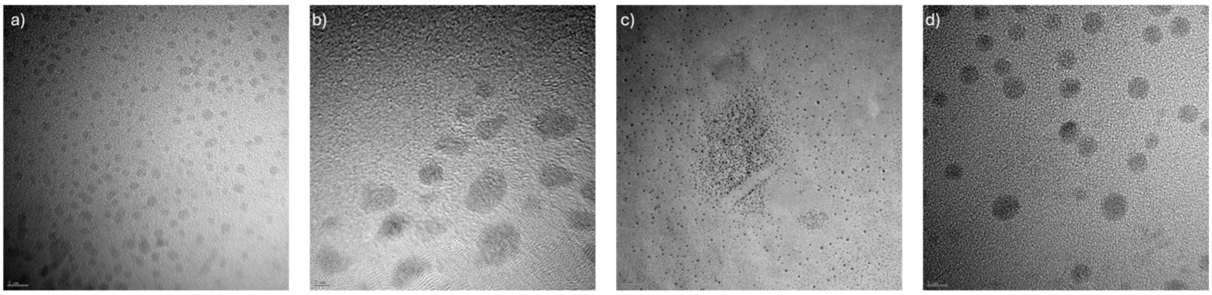


*Figure S11. TEM micrograph of particles synthesized at $AA/M^{2+}$ of around 1.3. a) and b) CoS. C) and d) $CoCO_3$.*

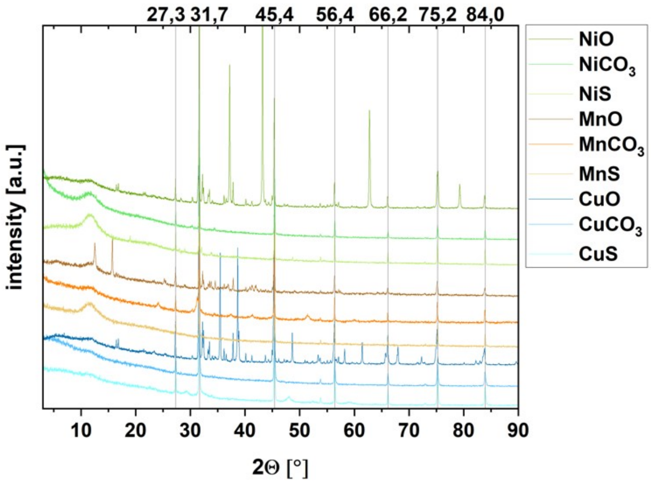


*Figure S12. XRD spectra of sulfide/carbonate/oxide particles derived from PAA/$M^{2+}$ coacervates for different cations. The visible reference lines and the reflexes visible in all spectra correspond to the crystal structure of NaCl residues. Also, visible the broad reflex expressing the presence of amorphous substance for $MnCO_3$, MnS, $NiCO_3$, and NiS. It is also slightly visible for $CuCO_3$ and CuS, but due to the synthesis at lower pH and therefore less produced particles, it is less expressed. For the calcinated carbonates reflexes appear and the intensity of the amorphous reflex decreases indicating the formation of ordered crystal structures.*

*Table S1. Mean radius (r) determined from TEM micrographs like those shown in Figure S6, Figure S7, and Figure S8 for nanoparticles of different metal cations with different anions. Carbonate and sulfide samples were obtained via from coacervate-forming solutions at pH 7 for $Mn^{2+}$ and $Co^{2+}$, at pH 8 for $Ni^{2+}$, and pH 4 for $Cu^{2+}$. Oxide particles were formed upon calcination of their carbonate counterparts. For each system, a statistically relevant number (n>50) of nanoparticles was analyzed, giving a reliable mean radius with corresponding standard deviations. Because of different morphologies, these particles can be separated by different sizes. Also shown, are the particle and aggregate sizes determined by dynamic light scattering. Average diameters were derived from the DLS data using the cumulants method* [19] *and the CONTIN algorithm* [21,22]*, which gives apparent sizes for both the primary particles and their aggregates. The values ($r_H$ < 2 nm) derived from CONTIN correspond to remains of the used PAA in the synthesis. Note that for the cumulants analysis, the first value depicts the suspension as it is, whereas the second value is created after filtration using a 200 nm CA membrane.*

| **Particle** | **r [nm] / TEM** | **$r_H$ [nm] / DLS(Cumulants)** | **$r_H$ [nm] / DLS(CONTIN)** |
|---|---|---|---|
| $CoCO_3$ | 4.81 ± 1.92<br>31.99 ± 9.69<br>171.85 ± 48.79 | 118.97 ± 79.53 (PD.I. 0.447)<br>32.61 ± 23.57 (PD.I. 0.522) | 0.927 (0.7%)<br>3.63 (11.2%)<br>32.6 (45.1%)<br>388 (40%) |
| CoO | 12.09 ±4.24<br>35.62 ± 11.47<br>351.08 ± 35.75 | 1379.21 ± 829.8 (PD.I. 0.362)<br>60.75 ± 28.68 (PD.I. 0.223) | 3.75 (0.4%)<br>54.1 (71.6%)<br>427 (22.2%) |
| CoS | 13.14 ± 5.03<br>63.19 ± 18.81<br>126.36 ± 21.99 | 214.25 ± 72.63 (PD.I. 0.115)<br>69.09 ± 41.24 (PD.I. 0.356) | 4 (1.3%)<br>61.5 (66.6%)<br>414 (30.2%) |
| $MnCO_3$ | 3.95 ± 0.97<br>38.58 ± 15.02<br>143 ± 17.93 | 143.08 ± 89.65 (PD.I. 0.393)<br>100.01 ± 64.96 (PD.I. 0.422) | 1.93 (0.7%)<br>67.6 (48%)<br>427 (47.7%) |
| MnO | 5.58 ± 2.73<br>188.81 ± 55.35 | 519 ± 361.21 (PD.I. 0.483)<br>2003.06 ± 1177.4 (PD.I. 0.346) | 16.2 (16.4%)<br>441 (83.6%) |
| MnS | 7.36 ± 4.31<br>19.65 ± 4.03<br>129.81 ± 17.22 | 130.32 ± 88.72 (PD.I. 0.463)<br>57.99 ± 27.89 (PD.I. 0.231) | 0.843 (0.03%)<br>29.6 (19%)<br>95.5 (76.7%) |
| $NiCO_3$ | 39.29 ± 10.78<br>247.62 ±124.78 | 99.47 ± 69.22 (PD.I. 0.484)<br>42.4 ± 30.03 (PD.I. 0.502) | 1.09 (0.9%)<br>6.24 (8.8%)<br>55.9 (58.7%) |

| | | | |
|---|---|---|---|
| | | | 364 (28.5%) |
| NiO | 73.81 ± 15.93<br>139.09 ± 44.41 | 222 ± 134.26 (PD.I. 0.366)<br>77.88 ± 30.18 (PD.I. 0.15) | 79.2 (84.3%)<br>414 (11.7%) |
| NiS | 9.17 ± 5.08<br>98.87 ± 12.23 | 139.9 ± 36.02 (PD.I. 0.0663)<br>41.87 ± 19.74 (PD.I. 0.222) | 0.957 (0.3%)<br>4.69 (0.4%)<br>54.1 (94.1%)<br>353 (1.1%) |
| $CuCO_3$ | | 5723.56 ± 3475.32 (PD.I. 0.369)<br>35.68 ± 27.06 (PD.I. 0.575) | 1.23 (10.1%)<br>7.31 (19.9%)<br>441 (73.6%) |
| CuO | | 419.24 ± 165.74 (PD.I. 0.156)<br>6145.34 ± 3666.75 (PD.I. 0.356) | / |
| CuS | | 220.1 ± 137.03 (PD.I. 0.388)<br>234.44 ± 150.99 (PD.I. 0.415) | / |
